# “We Also Had a Big Hit During COVID”: Realities of the COVID-19 Pandemic on Adult Day Services

**DOI:** 10.1093/geroni/igaf122.801

**Published:** 2025-12-31

**Authors:** Kingsley Udeh, Molly Noble, Heather Menne, Sara McLaughlin, Randi Hamill, MaKenna McClure, Laurinda Johnson

**Affiliations:** Miami University, Oxford, Ohio, United States; Miami University, Oxford, Ohio, United States; Miami University, Oxford, Ohio, United States; Miami University, Oxford, Ohio, United States; LeadingAge Ohio, Columbus, Ohio, United States; LeadingAge Ohio, Columbus, Ohio, United States; LeadingAge Ohio, Columbus, Ohio, United States

## Abstract

Adult day service (ADS) is a model of home-and-community-based services within the long-term care services and support continuum. ADS offers a system of professionally delivered, integrated, therapeutic, social, and health-related services in a structured and secure environment that helps individuals sustain living within the community. It also offers respite to family caregivers. There is growing need to understand the realities and struggles experienced by ADS providers, particularly in the years following the COVID-19 pandemic when many ADS programs were shuttered. The current study seeks to understand factors that made it easier or more difficult for providers to navigate through/after the pandemic. Seventeen ADS providers in Ohio participated in two focus groups. Transcripts were reconciled for accuracy and coded thematically to identify facilitators and barriers to service provision. Two key themes from the analysis include the importance of partnerships and education/awareness about ADS. Transcripts were further coded for constructs included in the Outer and Inner Setting domain of the updated Consolidated Framework for Implementation Science (CFIR). Outer and Inner setting domain constructs include external and internal influences that might affect organizational performance. Both facilitators and barriers to service provision fell primarily within the CFIR’s Outer setting, including critical incidence, local attitude and conditions, partnership and connections, and financing. Inner setting domain constructs included structural characteristics, relational connections, communications, and available resources. Study findings highlight the realities faced by ADS providers and highlight potential points of intervention for researchers and policy makers seeking to foster use and access to adult day services.

